# In-Situ Growth of Bimetallic ZnCo-ZIF-67 on Carbon Fibers as High-Efficiency Catalyst for Enhancing Thermal Decomposition of Ammonium Perchlorate

**DOI:** 10.3390/molecules31162767

**Published:** 2026-08-09

**Authors:** Junyu Li, Zhican Lu, Qihui Zeng, Fang Wang, Bo Yuan, Zeyu Zheng, Xiaolin Tang, Yifu Zhang, Chi Huang

**Affiliations:** 1 College of Chemistry and Molecular Sciences, Wuhan University, Wuhan 430072, China; junyu-li@whu.edu.cn (J.L.); zhicanlu@whu.edu.cn (Z.L.); 2023282030176@whu.edu.cn (B.Y.); zhengzeyu@whu.edu.cn (Z.Z.); 2 National Key Laboratory of Aerospace Chemical Power, Xiangyang 441003, China; zengqihui@whu.edu.cn (Q.Z.); wf198216@163.com (F.W.); 3 Hubei Institute of Aerospace Chemotechnology, Xiangyang 441003, China; 4 Hubei Key Laboratory of Radiation Chemistry and Functional Materials, School of Nuclear Technology and Chemistry & Biology, Hubei University of Science and Technology, Xianning 437100, China; yfzhang2023@whu.edu.cn

**Keywords:** in-situ growth, bimetallic catalyst, combustion, carbon fibers, ammonium perchlorate

## Abstract

Due to its abundant active sites, the bimetallic zeolite imidazole framework ZnCo-ZIF-67 exhibits excellent catalytic performance on the key oxidant ammonium perchlorate in composite solid propellants. In addition, carbon fiber has been proven to promote the combustion of propellants due to its high thermal conductivity efficiency. In order to integrate the advantages of both, this study designed and prepared a novel composite catalyst, ZnCo-ZIF-67/CF, by a co-precipitation method. The thermal decomposition test demonstrated that the ZnCo-ZIF-67/CF composite exhibited significant catalytic activity. When the addition amount was 5 wt%, the high-temperature decomposition peak temperature of AP decreased significantly from 424.3 °C to 337.2 °C, and the combustion process was also significantly accelerated. Furthermore, analysis of the products of thermal decomposition gases revealed a significant increase in the proportion of N_2_O in the catalyzed products to 55.7%, whilst the proportion of high oxidation state nitrogen-containing oxides such as NO_2_ and NOCl decreased. This finding suggests that the highly dispersed metal active sites in ZnCo-ZIF-67/CF synergistically promote the decomposition reaction pathway of AP, leading to enhanced N_2_O generation. This study proposes a novel approach for the development of efficient and stable AP decomposition catalysts, which has positive significance for the regulation of the combustion performance of propellants.

## 1. Introduction

Ammonium perchlorate (AP) has been identified as the most commonly used oxidant in composite solid propellants [1,2,3,4]. The thermal decomposition characteristics of the substance, including its decomposition temperature, rate, and gas product composition, directly affect the ignition performance, combustion rate, and energy release efficiency of the propellant [5,6]. Consequently, the regulation and acceleration of the thermal decomposition process of AP is identified as a pivotal strategy to enhance the combustion performance of solid propellants [7,8,9]. In the course of the research, a number of approaches were explored. The incorporation of catalysts was identified as a particularly effective and viable method. Transition metal oxides (e.g., CuO [10,11], Cu_2_O [12], Fe_2_O_3_ [13,14], Co_3_O_4_ [15,16], etc.) have attracted considerable attention due to their significant catalytic activity in the decomposition of AP. It has been demonstrated that these substances are capable of reducing the decomposition temperature of AP and accelerating the reaction process. However, nanoscale transition metal oxides face a common and severe challenge in practical applications: namely, particle aggregation. The agglomeration phenomenon leads to a sharp decrease in the effective specific surface area of the catalyst, resulting in insufficient exposed active sites and severely limiting its catalytic efficiency. Consequently, the primary concern in enhancing the efficiency of AP catalytic decomposition is the method by which to achieve highly dispersed and stable loading of catalyst nanoparticles while ensuring optimal mass and heat transfer conditions.

In recent years, metal–organic framework materials (MOFs) [6,17], especially zeolite imidazolate frameworks (ZIFs) [18,19], have demonstrated considerable potential in the field of catalysis due to their ultra-high specific surface area, adjustable pore structure, and abundant metal active centres. Among them, bimetallic ZIFs (e.g., ZnCo-ZIF-67) frequently demonstrate superior catalytic performance in comparison to single metal MOFs [20].

Meanwhile, the exceptional thermal and electrical conductivity of carbon fibre functions as an efficient electron transfer conduit for the catalytic reaction process, thereby enhancing electron transfer efficiency and augmenting catalytic efficiency [21,22]. Furthermore, the chemical inertness and thermal stability of carbon fibre contribute to its remarkable durability in high-temperature catalysis and corrosive medium reactions, thus rendering it an optimal catalyst carrier [23,24,25]. In addition, the high thermal stability and good thermal conductivity of carbon fibre itself have been proven to have a beneficial effect on the combustion of propellants [26,27]. Consequently, the utilisation of carbon fibre as a catalyst carrier for propellants is anticipated to fulfil a multitude of functions during the combustion process of propellants.

Therefore, this article synthesized a composite catalytic material, ZnCo-ZIF-67/CF, using a co-precipitation method. It is supported by carbon fibers and combined with bimetallic ZIF. It is expected to effectively reduce the decomposition temperature of AP and improve the decomposition rate of AP, solving the challenges of catalyst aggregation and limited heat transfer in the system. A series of analytical methods, including SEM, TEM, XRD, and XPS, were used to systematically characterize the morphology, structure, and composition. On this basis, the catalytic effect of composite materials on the thermal decomposition behavior of AP was deeply studied, and its catalytic effect was analyzed using TG-DSC. The evolution of decomposed gas products was monitored through TG-IR, and possible catalytic mechanisms were explored. The aim of this study is to provide new material design and preparation strategies for the development of high-performance AP decomposition catalysts, and to provide experimental evidence for understanding their catalytic mechanisms.

## 2. Results and Discussion

### 2.1. Synthesis and Characterization of ZnCo-ZIF-67/CF

**Figure 1:**
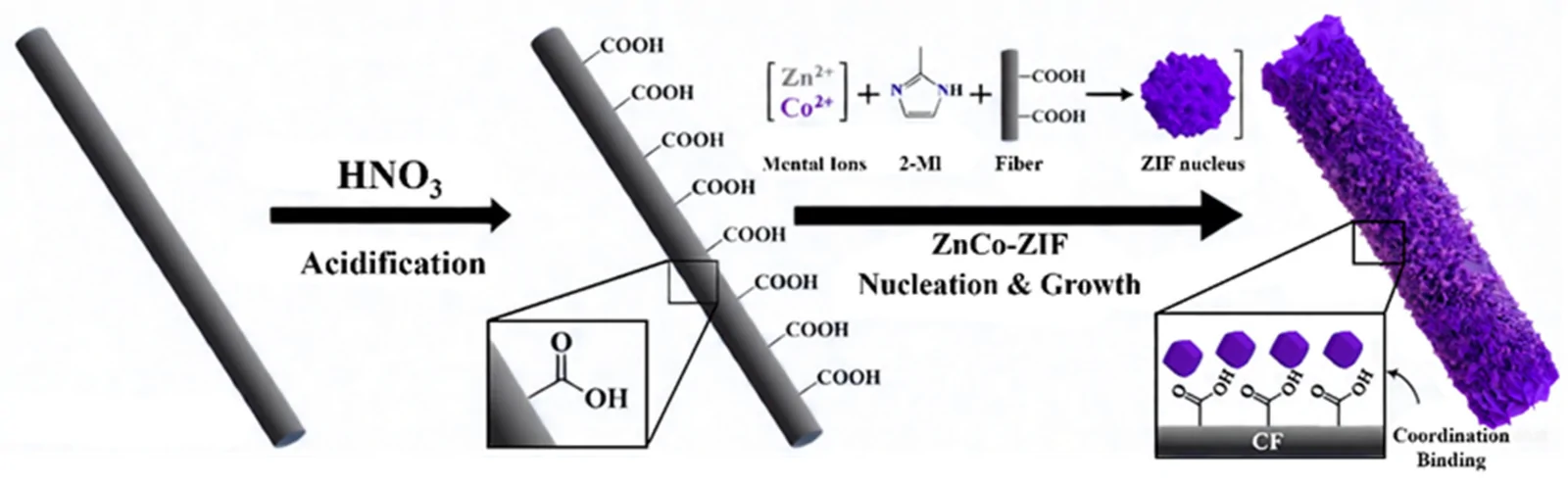
Schematic diagram of the synthesis of ZnCo-ZIF-67/CF.

The loading of ZnCo-ZIF-67 on carbon fibres was achieved using the method demonstrated in Figure 1, resulting in the formation of ZnCo-ZIF-67/CF. A universal method is employed in the initial phase for the purpose of removing the sizing agent from the surface of carbon fibres. Subsequently, the hydroxyl groups present on the surface of carbon fibres CF-OH undergo oxidation to form carboxyl groups CF-COOH through the process of concentrated nitric acid oxidation. It has been proven that carboxyl groups have certain chelating properties when interacting with metal ions. This facilitates the formation of nucleation sites on the surface of carbon fibres. Subsequently, the carbon fibre was immersed in a solution that contained metal ions. The purpose of this step is to give sufficient time for chelation to occur. Finally, the incorporation of ligands was undertaken to synthesise samples of ZnCo-ZIF-67/CF, which were subsequently loaded onto the carbon fibre through the process of hydrothermal reaction.

**Figure 2:**
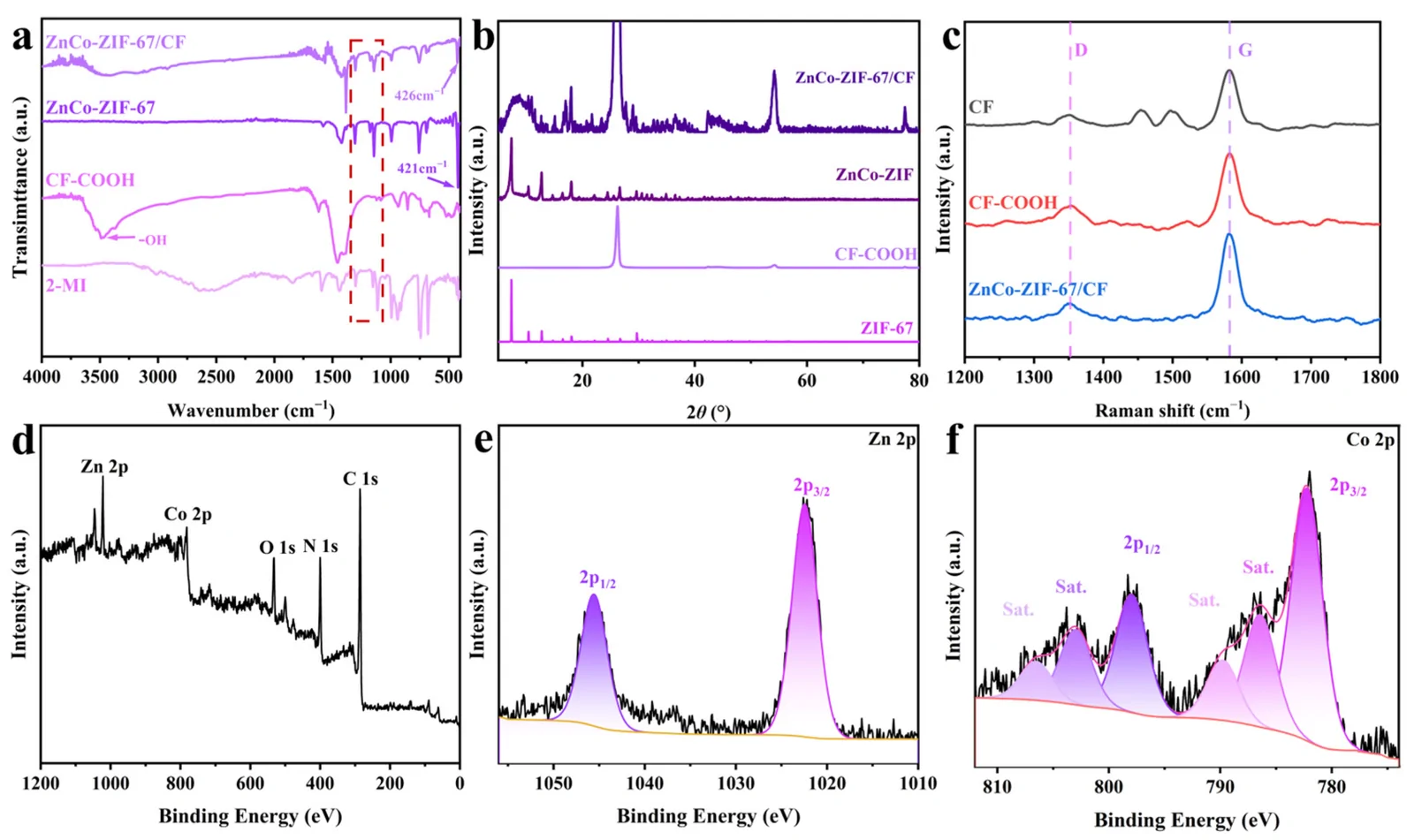
Characterization results of ZnCo-ZIF-67/CF: (**a**) FT-IR (The dashed line represents the characteristic peak of imidazole); (**b**) XRD; (**c**) Raman (D represents D band, G represents G band); (**d**) XPS; (**e**) Zn 2*p*; (**f**) Co 2*p*.

The alterations in functional groups on the surface of carbon fibres, along with the successful loading of ZnCo-ZIF-67, were examined by infrared spectroscopy [28]. The test results are presented in Figure 2a. The surface of CF-COOH displays a prominent -OH absorption peak at 3500 cm^−1^, attributable to the presence of a substantial number of carboxyl groups and unoxidized hydroxyl groups. In addition, a clear carbonyl absorption peak is visible at 1700 cm^−1^, and a strong graphitization peak is also observed at 1500 cm^−1^. In the case of ZnCo-ZIF-67, the coordination of 2-methylimidazole (2-MI) results in a shift of the absorption peak to varying degrees, with the appearance of a clear M-N (M=Zn, Co) absorption peak at 421 cm^−1^. For ZnCo-ZIF-67/CF, the characteristic peaks are indicative of both ZnCo-ZIF-67 and CF-COOH, thus confirming the successful loading of ZnCo-ZIF-67 on carbon fibres.

Furthermore, the structure of ZnCo ZIF in the synthesised product was verified by XRD, as illustrated in Figure 2b. For CF-COOH, a single peak is observed, indicative of the characteristics typically associated with carbon materials [29]. For ZnCo-ZIF-67, distinct diffraction peaks are exhibited at 2*θ* = 11.3°, 13.7°, 15.8°, 17.5°, etc. The positions of these diffraction peaks are consistent with the characteristic peaks of ZnCo-ZIF-67 reported in previous literature [30,31], indicating that the ZnCo-ZIF-67 structure has been successfully synthesised. These characteristic peaks also exist in ZnCo-ZIF-67/CF. In combination with the discernible carbon fibre peak at 2*θ* = 25.0°, this observation serves to substantiate the coexistence of ZnCo-ZIF-67 and carbon fibre structures within the specimen.

The presence of defects in the carbon fibres was analysed using Raman spectroscopy, and the test results are displayed in Figure 2c. It is evident that all samples exhibit characteristic peaks at 1350 cm^−1^ (D band) and 1590 cm^−1^ (G band). This indicates that the carbon material is composed of graphitized carbon and non-graphitized carbon. However, given that the carbon fibres utilised in this study are high-modulus carbon fibres with a high degree of graphitization, the peak area of the G band is significantly larger than that of the D band. For CF-OH, its I_D_/I_G_ is only 0.15. Following the acidification process, the I_D_/I_G_ increases to 0.25, given the inherent susceptibility of graphite to acidification. Consequently, as a consequence of the ZnCo-ZIF-67 loading concentration on the acidified sites, the I_D_/I_G_ was restored to 0.16.

Furthermore, in order to illustrate the elemental composition of the ZnCo-ZIF-67/CF surface, XPS testing was performed on the sample, and the test results are shown in the Figure 2d–f and Figure S1 [30]. The full-spectrum test results of ZnCo-ZIF-67/CF show the presence of signals such as C 1*s*, N 1*s*, O 1*s*, Co 2*p*, and Zn 2*p*, which constitute the basic framework of ZnCo-ZIF-67. In this study, the photoelectron peaks of Co at 2*p*^3/2^ and 2*p*^1/2^ were observed at 782.24 eV and 797.95 eV, respectively. In addition, due to the simultaneous presence of Co^2+^ and Co^3+^ in ZnCo ZIF, the satellite peak significantly widens and can split into two small peaks. Furthermore, the photoelectron peaks of 2*p*^3/2^ and 2*p*^1/2^ of Zn^2+^ were observed at 1021.86 eV and 1045.06 eV, respectively. This outcome aligns with the findings documented in pertinent literature, thereby substantiating the efficacy of the synthesis process through the aforementioned analytical method.

On the basis of the above tests, in order to further confirm the content of ZnCo-ZIF-67 in the ZnCo-ZIF-67/CF sample, the sample was digested with nitric acid and ICP was tested. The test results are shown in Table S1. According to the test results, the calculated content of ZnCo-ZIF-67 in the sample is 17.52% ± 0.04%, which further indicates that the effective loading of catalytic components on carbon fibers has been achieved through the synthesis method.

Unfortunately, due to the interference of carbon fiber itself in adsorption testing, it is difficult to obtain normal adsorption data. Therefore, this article does not provide information on the material’s adsorption performance.

**Figure 3:**
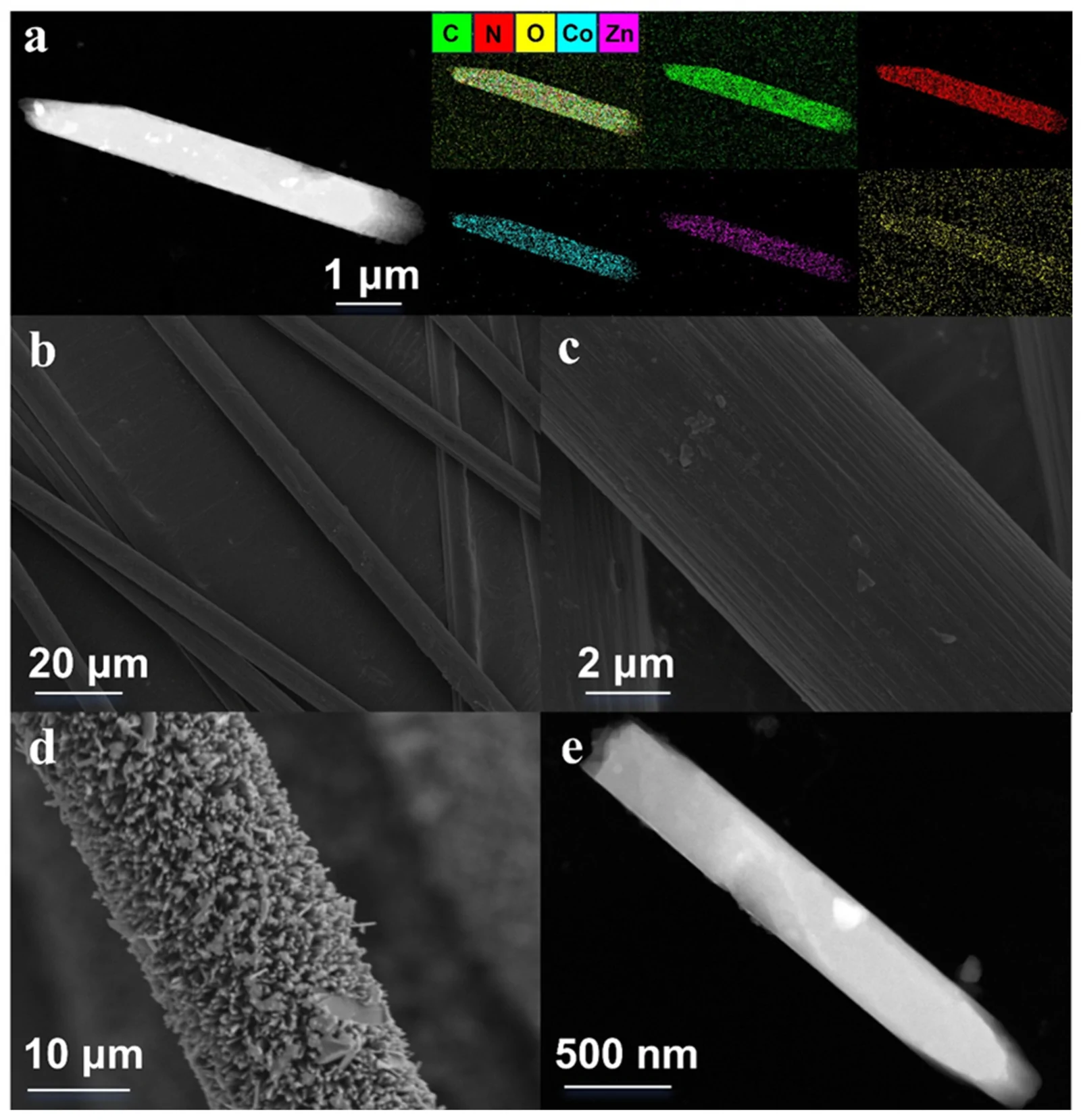
Morphology characterization test results: (**a**) TEM mapping of ZnCo-ZIF-67 on carbon fiber; (**b**) SEM image of CF-OH; (**c**) SEM image of CF-COOH; (**d**) SEM image of ZnCo-ZIF-67/CF; (**e**) TEM image of ZnCo-ZIF-67 on carbon fiber.

Furthermore, in order to observe more intuitively whether ZnCo-ZIF-67 has been successfully loaded on the surface of carbon fibres, SEM and TEM were used to observe the morphology of samples at different stages, and the test results are shown in Figure 3 and Figures S2–S4. The surface of CF-OH is characterised by relative smoothness, with a diameter of carbon fibres measuring below 10 μm. Upon oxidation to CF-COOH, a transformation in surface texture is evident, accompanied by negligible alterations in macroscopic morphology. For the ZnCo-ZIF-67/CF sample, a significant number of particles appeared on the surface of the carbon fibre, and the particles were almost completely loaded onto the entire surface of the carbon fibre, thus proving the successful loading of ZnCo-ZIF-67 on the carbon fibre. It was observed that the loading of particles resulted in an increase in the diameter of the carbon fibre structure to over 10 μm. The EDS images also prove that ZnCo-ZIF-67 is heavily loaded on the surface of carbon fibers. Furthermore, TEM observation was conducted on the ZnCo-ZIF-67 structure loaded on the surface of carbon fibres, and it was determined that the loaded ZnCo-ZIF-67 exhibited block or flake-like structures. Furthermore, the mapping results indicated that the flake-like structure was distributed uniformly with Zn, Co, C, N, and O elements.

In order to elucidate the structural changes of catalyst components during the catalytic process, thermogravimetric experiments were conducted on carbon fibers, ZnCo-ZIF-67, and ZnCo-ZIF-67/CF in an air atmosphere (Figure S5). The experimental results indicate that due to the main structure of carbon fiber being graphite, the weight loss of carbon fiber samples is relatively small at lower temperatures, but oxidation weight loss occurs at higher temperatures. The weight loss process shows a certain degree of correlation with the surface structure, which may be due to the damage caused by the treatment process to the surface structure, making it more susceptible to O_2_ attack. For ZnCo-ZIF-67, the weight loss stage is approximately 450 °C, after which the material remains stable. The early decomposition temperature of ZnCo–ZIF-67/CF synthesized in this article is consistent with that of ZnCo-ZIF-67, which confirms the existence of this structure. However, in the later stage, the decomposition rate significantly increased, and even the residual amount was lower than that of CF-COOH and ZnCo-ZIF-67. This phenomenon may be attributed to the catalytic oxidation of carbon fibers by the oxides generated during the decomposition process of ZnCo-ZIF-67.

### 2.2. Analysis of the Effect of AP Catalysed by ZnCo-ZIF-67/CF

**Figure 4:**
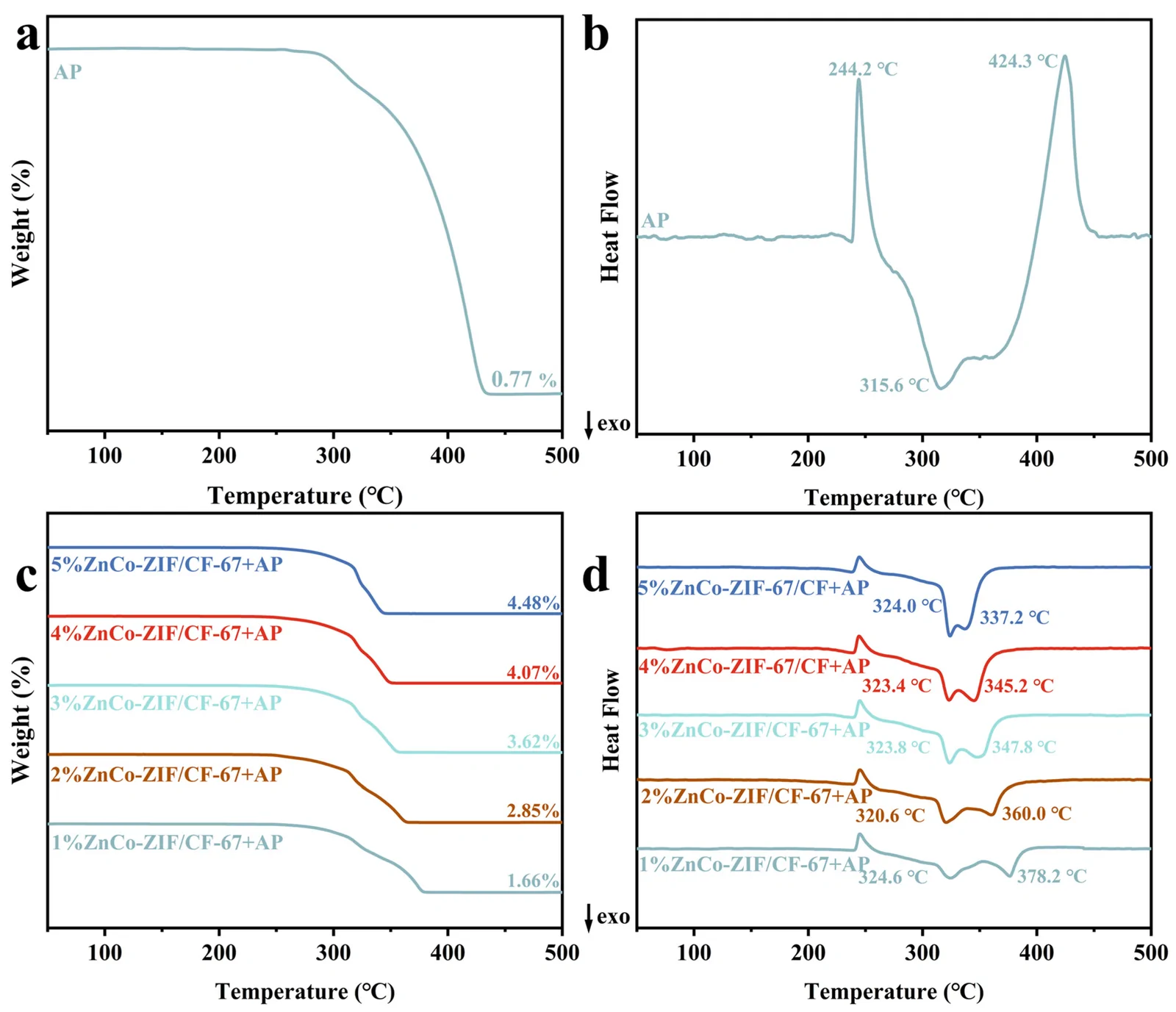
TG-DSC test results of AP itself and ZnCo-ZIF-67/CF catalyzed AP decomposition: (**a**) TG of AP itself (numbers represent residual weight); (**b**) DSC of AP itself; (**c**) TG of ZnCo-ZIF-67/CF catalyzed AP (numbers represent residual weight); (**d**) DSC of ZnCo-ZIF-67/CF catalyzed AP.

Following a comprehensive characterisation of the properties of ZnCo-ZIF-67/CF material, its catalytic performance for AP decomposition was examined, and the test results are presented in the Figure 4. As illustrated in Figure 4a,b, the thermal decomposition of AP follows a three-stage process, consistent with the findings in the extant literature. This includes a crystal transformation stage at 244.2 °C. The transformation of AP from its original orthorhombic crystal system to a cubic crystal system is an endothermic process. In the low-temperature decomposition stage (around 315.6 °C), AP dissociates into HClO_4_ and NH_3_ through proton transfer, which is an exothermic process. The process of diffusion of NH_3_ is gradual, while the adsorption of HClO_4_ on the surface of AP is inhibitive to subsequent decomposition reactions, thus entering a “stagnation” stage. With an increase in temperature, the desorption of HClO_4_ from the surface of AP initiates the restart of the decomposition reaction at elevated temperatures, followed by subsequent redox reactions. This ultimately results in a complete reaction and the formation of a high-temperature decomposition peak near 424.3 °C.

As demonstrated in Figure 4c,d and Table S2, the doping of ZnCo-ZIF-67/CF has been shown to catalyse the thermal decomposition process of AP with high efficiency. The findings demonstrated that when the mixing ratio was set at 5%, the peak temperature of AP high-temperature decomposition decreased from 424.3 °C to 337.2 °C. Furthermore, the catalytic effect exhibited a gradual enhancement in conjunction with the increase in catalyst addition, which can be attributed to the augmentation in the number of catalytic active sites.

For the reason of good catalytic effect, this article speculates that ZnCo-ZIF-67/CF, as a catalyst for AP decomposition, plays multiple roles in the AP decomposition process. On the one hand, AP decomposition leads to the formation of an oxygen-rich environment, which rapidly decomposes ZnCo-ZIF-67/CF into corresponding metal oxides. The metal centers in the metal oxides have been identified as active sites for the oxidation–reduction of gas-phase products formed by AP dissociation, thus exhibiting good catalytic effects. In addition, the oxidation of ligands requires a large amount of reactive oxygen species, which further promotes the decomposition of intermediate HClO_4_, which can also promote the decomposition of AP. As for carbon fiber, its function is more expected to be reflected in enhancing the thermal conductivity of the system after adding propellant, while in the process of AP decomposition, it can only promote the initial decomposition of AP through its own conductivity. This is demonstrated by the data of CF-COOH on the catalytic activity of AP, as shown in Figure S6. Although the catalytic effect of CF-COOH on AP is relatively weak, it still indicates that CF-COOH can enhance the initial decomposition of AP through electron transfer mechanism.

It should be noted that the results of pure ZnCo-ZIF-67 catalyzing AP have been proposed in our previous work [20]. After comparison, it can be concluded that there is no significant difference in catalytic performance under a 5% load, and the position of the high-temperature decomposition peak is basically the same. Therefore, it can be inferred that the use of carbon fiber support will not have a substantial negative impact on the catalytic performance of ZnCo-ZIF-67, but the addition of carbon fiber can play a functional role in subsequent propellant applications.

On this basis, in order to clarify the authenticity of the above catalytic mechanism speculation, this article analyzed the products of the catalyst calcined in air to simulate the residue of the catalyst after high-temperature decomposition in a strong oxidizing atmosphere. The test results are shown in Figures S7–S9. The XRD results show that the decomposed product is mainly Co_3_O_4_, while the residual amount of ZnO is relatively small, which is consistent with the elemental content analysis obtained from ICP testing. The infrared spectroscopy results show that the organic components in the product have been largely oxidized, making it difficult to observe the characteristic peaks of organic compounds. The SEM results show that the overall product can still maintain its rod-shaped structure, but due to the decomposition of the ZIF structure, the rod-shaped structure is actually composed of many micrometer-sized sheet-like structures. Combined with XRD and EDS results, these sheet-like results are speculated to be Zn-doped Co_3_O_4_ materials grown after decomposition. It should be noted that although ZnCo-ZIF-67/CF-C was used instead of the catalyst sample after catalyzing AP in this article, there are still acceptable differences between the two samples. Because there are very few residual samples after catalysis, obtaining sufficient samples requires a lot of AP consumption, which poses a danger. Therefore, sample substitution was carried out.

In order to demonstrate the superior advantages of ZnCo-ZIF-67/CF in catalyzing AP compared to ZnCo-ZIF-67/CF-C, which has already become an oxide, the performance data of ZnCo-ZIF-67/CF-C in catalyzing AP were tested. The test results are shown in Figure S10, which shows a significant decrease in its catalytic performance. After analysis, it is believed that pure oxides cannot promote the decomposition of AP by consuming reactive oxygen species due to the absence of organic structures in their structure. On the other hand, the original grains generated by in-situ decomposition may have smaller sizes and larger specific surface areas, resulting in higher catalytic activity, while fully grown oxides have obvious disadvantages and therefore poorer catalytic performance.

### 2.3. Analysis of the AP Product Catalysed by ZnCo-ZIF-67/CF

**Figure 5:**
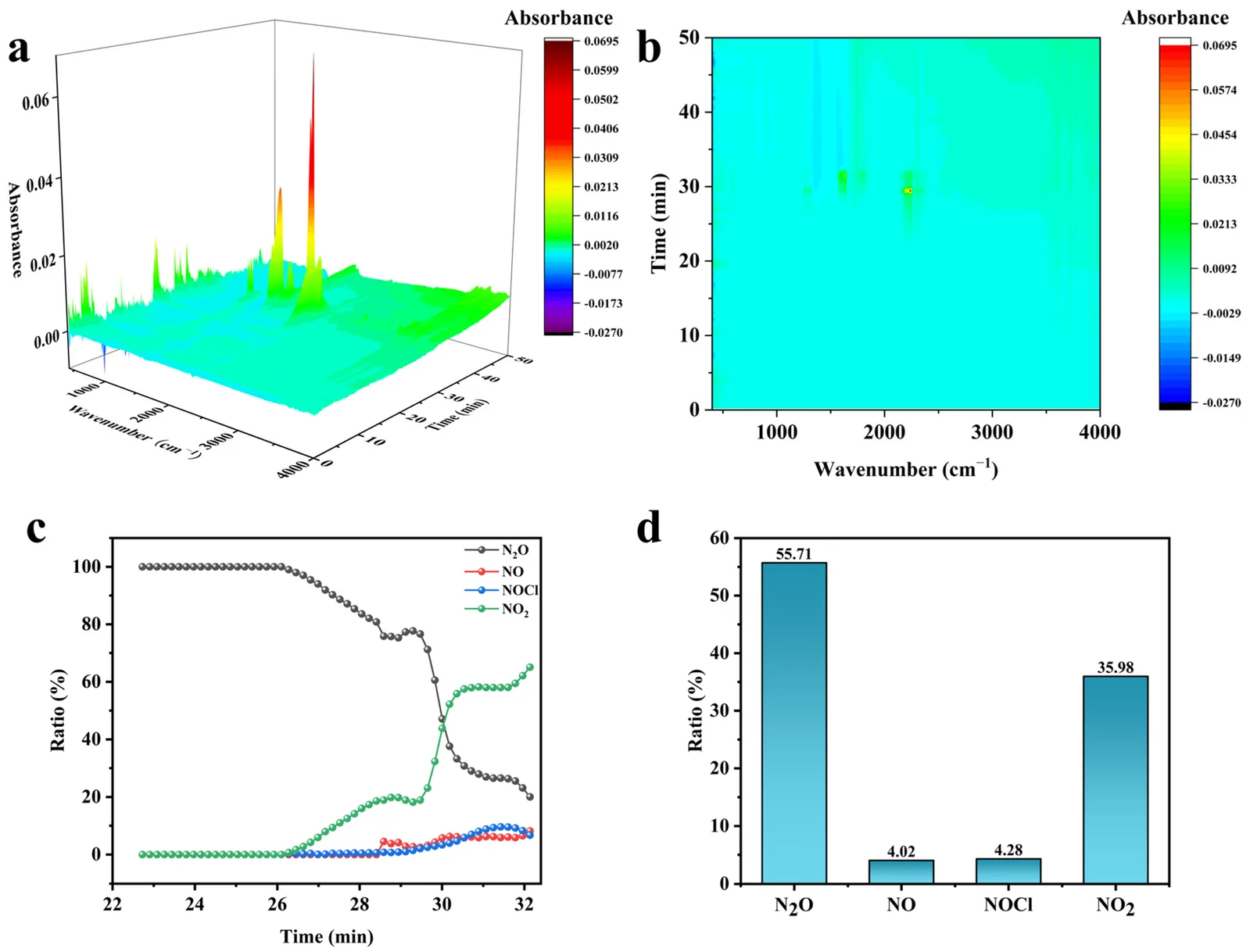
Gas composition analysis of ZnCo-ZIF-67/CF catalyzed AP decomposition: (**a**) TG-IR diagram; (**b**) TG-IR mapping diagram; (**c**) gas composition variation over time; (**d**) nitrogen containing gas ratio diagram without N_2_.

Furthermore, the alterations in gas products during the decomposition process of AP before and after the addition of catalyst were examined, and the results are displayed in Figure 5 and Figure S11. Generally, the main process of AP decomposition (especially ammonia oxidation) is as follows:

HClO_4_ → ·ClO_3_ + ·OH

·OH + NH_3_ → ·NH_2_ + H_2_O

·ClO_3_ + NH_3_ → ·NH_2_ + HClO_3_

HClO_4_ + ·NH_2_ → ·H_2_NO + HClO_3_

·H_2_NO + ·OH → HNO + H_2_O

·H_2_NO + ·ClO_2_ → HNO + HClO_2_

·H_2_NO + ·ClO → HNO + HClO

2HNO → N_2_O + H_2_O

4HNO + O_2_ → 4NO + 2H_2_O

2NO + Cl_2_ → 2NOCl

2NO + O_2_ → 2NO_2_

In previous research, a methodology for analysing the decomposition process of AP through changes in nitrogen oxides (including N_2_O, NO, NOCl, NO_2_) was proposed [32]. In the initial low-temperature decomposition stage of pure AP, the primary oxidation product of NH_3_ is N_2_O (excluding N_2_ without infrared signal), accompanied by the production of NO_2_ in the middle and later stages of this stage. During the high-temperature decomposition stage, there is a significant increase in the content of NO_2_, and the gas released by AP decomposition becomes nitrogen oxide gas, which is mainly composed of NO_2_ and N_2_O. It can be deduced from the gas generation during the low-temperature and high-temperature decomposition stages that the active oxygen content in the gas phase is lower during the low-temperature decomposition stage. This results in NH_3_ being oxidized to lower valence states, N_2_O and N_2_, in the gas phase. In the high-temperature decomposition stage, an increase in the content of active oxygen species was observed in comparison with the low-temperature decomposition stage. This increase in active oxygen species enabled NH_3_ to be more oxidized to higher valence states, NO, NOCl, and NO_2_. As is evident from the analysis of the AP decomposition process, the aforementioned phenomenon can be attributed to the fact that HClO_4_, which is generated by the dissociation of AP during the low-temperature decomposition stage, is mainly adsorbed on the defect sites of AP. This results in a greater quantity of NH_3_ present in the gas phase during the low-temperature decomposition stage, with a comparatively lower amount of active oxygen species.

The TG-IR test results of the mixture of AP and 5 wt% ZnCo-ZIF-67/CF were analysed, and it was found that the composition of gas products during the entire decomposition process was basically similar to that of AP. However, compared with the gas composition of AP decomposition at the same temperature, it can be found that the proportion of high-oxidation-state gases, such as NO_2_, will significantly increase. This represents that under the action of the catalyst, the N-containing gas is more fully oxidized. This may also be the catalytic mechanism of the catalyst for the decomposition of AP; that is, by promoting the decomposition of HClO_4_ to release sufficient active oxygen to oxidize NH_3_, on the one hand, it shifts the reaction equilibrium of AP conversion to perchloric acid and ammonia to the right. On the other hand, it significantly accelerates the conversion rate of nitrogen-containing gases such as NH_3_ to oxidized nitrogen oxides, while releasing more heat to accelerate the reaction. Moreover, carbon fibers themselves have excellent thermal conductivity, which is undoubtedly beneficial for the decomposition process of AP. However, due to the catalytic effect of the catalyst itself, the decomposition reaction is completed at a lower temperature, which leads to a similar composition of the decomposition products as a whole, although in reality, their decomposition processes are completely different.

### 2.4. Kinetic Analysis of the AP Reaction Catalysed by ZnCo-ZIF-67/CF

**Figure 6:**
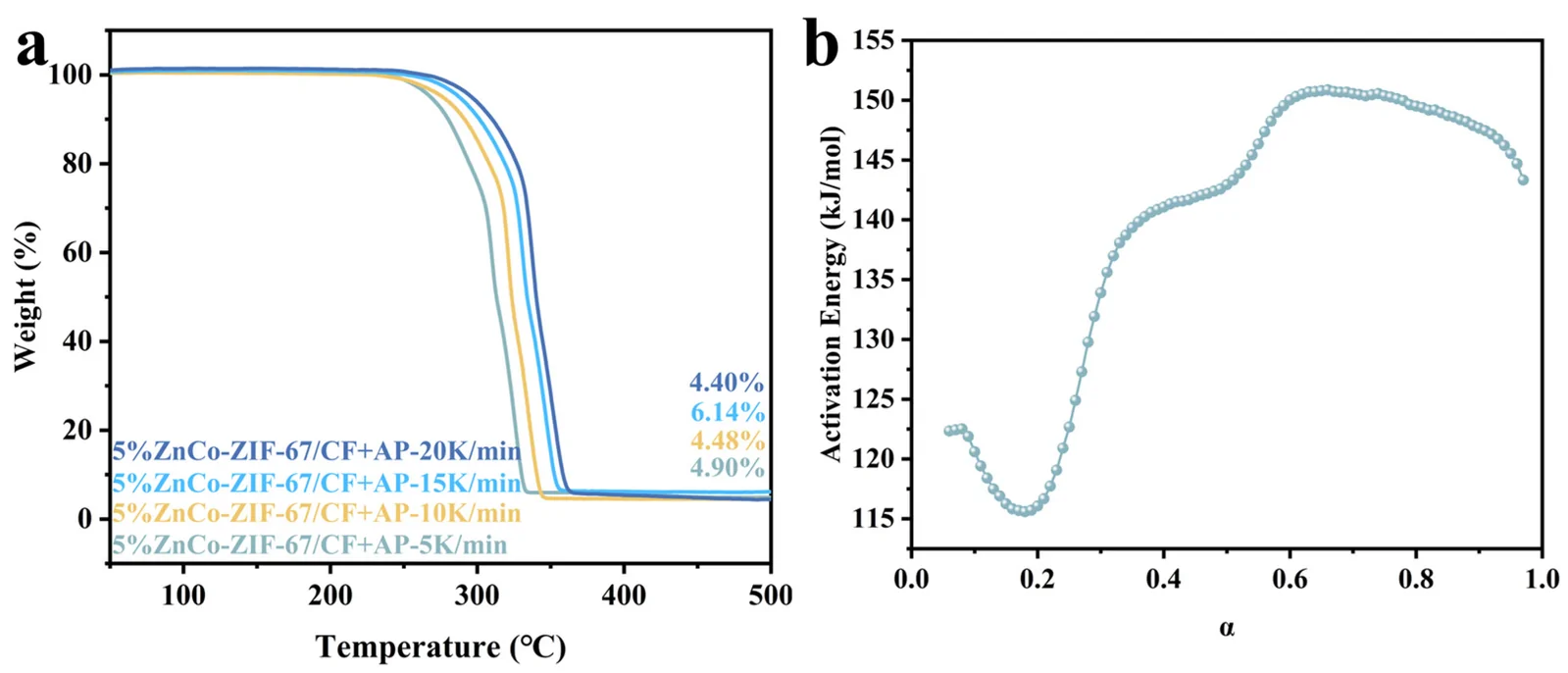
Dynamics analysis of ZnCo-ZIF-67/CF catalyzed AP: (**a**) TG of ZnCo-ZIF-67/CF catalyzed AP at different heating rates (numbers represent residual weight). (**b**) Variation in activation energy of ZnCo-ZIF-67/CF catalyzed AP with reaction degree.

In order to calculate the change in activation energy of AP decomposition process before and after catalyst addition, the thermogravimetric curves of AP were tested at different heating rates, and the test results are shown in Figure 6 and Figure S12 [33,34]. For AP samples, the trend of activation energy changes demonstrates a pattern of first decreasing and then increasing, and the degree of reaction at the extreme point is essentially consistent with the position where AP stagnates in the low-temperature decomposition stage. This finding also suggests that AP undergoes corresponding changes in its rate step when transitioning from the low-temperature decomposition stage to the high-temperature decomposition stage. Specifically, within the AP decomposition process, the gas desorption process on the surface of the AP crystal replaces the AP decomposition process and becomes the decisive step in the reaction. As the temperature is increased, the process of AP decomposition into HClO_4_ and NH_3_ once again becomes the pivotal step.

The activation energy during the decomposition of AP catalyzed by ZnCo ZIF/CF shows a trend of first decreasing and then increasing, followed by a gradual decrease. A comparison of the activation energy of AP itself reveals that the change pattern of activation energy throughout the process is consistent, with only numerical differences. Consequently, the findings of the present study serve to corroborate the preceding hypotheses concerning the catalytic process. That is to say, the catalyst merely expedites the oxidation–reduction reaction between AP decomposition derivatives by means of its own adsorption of gas, exerting minimal influence on the reaction pathway.

### 2.5. Combustion Process of the AP Reaction Catalysed by ZnCo-ZIF-67/CF

**Figure 7:**
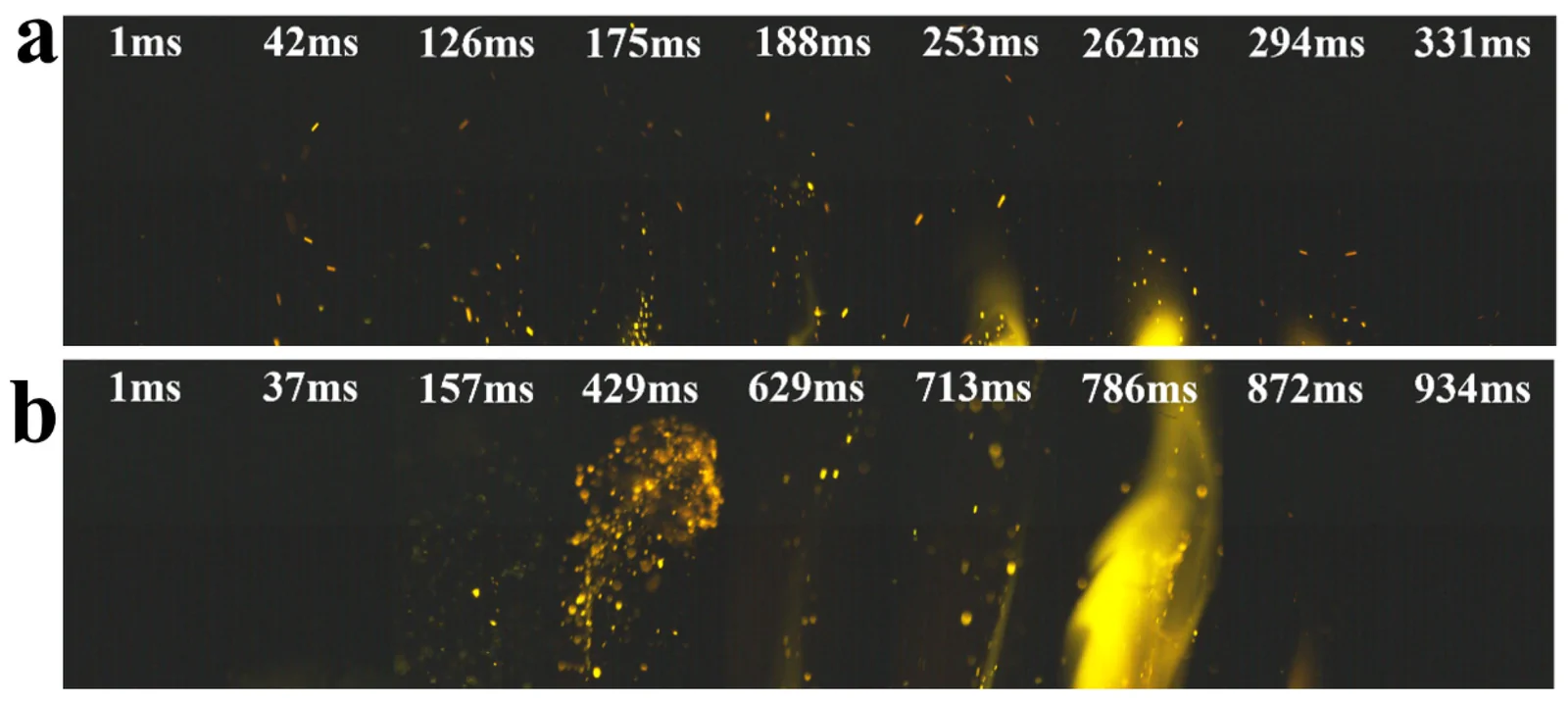
Combustion process photos of Samples: (**a**) AP with 5% ZnCo-ZIF-67/CF; (**b**) AP.

In order to observe and study the effect of ZnCo-ZIF-67/CF on the combustion performance of AP, laser ignition combustion experiments were conducted. The results of this testing are shown in Figure 7. The combustion process of AP itself is consistent with the reported results in relevant literature. The process was protracted, and only yellow flames became visible in the later stage. In the initial phase, the flames were characterised by a paucity of thermal energy, with a substantial quantity of AP particles being expelled into the gas phase as a consequence of gas action. The addition of the catalyst to the AP combustion process has been shown to result in a substantial reduction in the flame appearance time, leading to a significant acceleration in the reaction and disappearance rate of particles in the gas phase. This finding suggests that the catalyst developed in this study offers significant advantages in terms of accelerating AP decomposition.

## 3. Materials and Methods

### 3.1. Materials

All reagents were purchased from commercial sources and used without further purification. Zinc nitrate hexahydrate (Zn(NO_3_)_2_·6H_2_O, 99%), cobalt nitrate hexahydrate (Co(NO_3_)_2_·6H_2_O, 99%), 2-methylimidazole (C_4_H_6_N_2_, 2-MI, AR), ethanol (CH_3_CH_2_OH, AR), acetone (CH_3_COCH_3_, AR) used in this study were all purchased from Sinopharm Chemical Reagent Co., Ltd. (Shanghai, China). T-800 carbon fiber used in this study was all purchased from Guangwei Composite Co., Ltd. (Weihai, China). Distilled water is self-made in the laboratory. More Materials and Methods details in Supplementary Materials. The detailed equipment is also presented in Supplementary Materials.

### 3.2. Synthesis

Pre-treatment of carbon fiber: T-800 carbon fibers were soaked in acetone for 2 h to remove the sizing agent attached to the fiber surface. After soaking, the carbon fibers were transferred to a round-bottom flask, and concentrated nitric acid (≥68%) was added, followed by treatment under constant stirring at 70 °C for 4 h. Upon completion of the reaction, the fibers were rinsed repeatedly with deionized water until neutral and then vacuum-dried at 60 °C to constant weight. The obtained activated carbon fibers were designated as CF-COOH.

Preparation of ZnCo-ZIF-67/CF: First, 0.83 mmol of Zn(NO_3_)_2_·6H_2_O and 1.66 mmol of Co(NO_3_)_2_·6H_2_O were dissolved in 20 mL of deionized water and stirred for 5 min to obtain a pink solution A. Meanwhile, 20.0 mmol of 2-methylimidazole was dissolved in 20 mL of deionized water to obtain solution B. While stirring, solution A was slowly added to solution B. After thorough mixing, the mixed solution was transferred to the inner lining of a 100 mL hydrothermal reaction vessel, and the CF was completely immersed in the mixed solution. The vessel was heated at 60 °C for 4 h. Subsequently, the sample was taken out, washed three times with deionized water, and vacuum-dried at 50 °C to afford ZnCo-ZIF-67/CF with a purple surface.

Preparation of ZnCo-ZIF-67: Next, 0.33 mmol of Zn(NO_3_)_2_·6H_2_O and 0.66 mmol of Co(NO_3_)_2_·6H_2_O were dissolved in 20 mL of deionized water and stirred for 5 min to obtain a pink solution A. 0.75 mmol of 2-methylimidazole was dissolved in 20 mL of deionized water to obtain solution B. Solution A was slowly added to solution B while stirring, and after thorough mixing, the mixed solution was transferred to the inner lining of a 100 mL hydrothermal reactor. The vessel was heated at 60 °C for 4 h. The sample was then taken out, washed three times with deionized water, and vacuum-dried at 50 °C to obtain ZnCo-ZIF-67.

Preparation of ZnCo-ZIF-67/CF-C: The ZnCo-ZIF-67/CF precursor was placed in an alumina crucible and transferred into a tube furnace. Subsequently, the sample was heated from ambient temperature to 800 °C in an air atmosphere at a ramp rate of 10 °C·min^−1^. After natural cooling to room temperature, the crucible was taken out, and the resulting product was denoted as ZnCo-ZIF-67/CF-C.

Preparation of AP/catalyst mixture: An appropriate amount of catalyst with AP was mixed and ethanol was added. After ultrasonic treatment, dry at 40 °C. Then mix the dried powder thoroughly until there is no obvious color difference in each area. Unless otherwise specified in this article, the mass fraction of the catalyst is 5%.

## 4. Conclusions

This study successfully prepared bimetallic ZnCo-ZIF-67/CF composites using acidified carbon fiber (CF-COOH) as the substrate through a co-precipitation method, and systematically investigated their catalytic performance for AP thermal decomposition process. Based on the above experimental analysis and discussion, the following conclusions can be drawn:

(1) The characterization results indicate that ZnCo-ZIF-67 successfully grows on the surface of carbon fibers and ultimately becomes a composite structure interwoven with nanosheets. This structure significantly increases the specific surface area of the composite material and the effective contact interface with AP particles, providing a good morphological basis for the subsequent improvement of catalytic performance.
(2) The catalytic decomposition test results showed that after adding 5% mass fraction of ZnCo ZIF/CF, the high-temperature decomposition peak temperature of AP decreased from 424.3 °C to 337.2 °C (the exothermic peak advanced by 87.1 °C). The significant enhancement of catalytic activity can be attributed to the synergistic coupling effect between the highly dispersed metal active sites in ZnCo-ZIF-67 and the excellent thermal/electrical conductivity of carbon fibers.
(3) The results of thermogravimetric infrared spectroscopy (TG-IR) analysis and the calculation of apparent activation energy for decomposition indicate that the catalytic function of ZnCo-ZIF-67/CF essentially stems from its accelerating effect on the kinetics of redox reactions between intermediate products in the AP decomposition process. In addition, under this catalytic pathway, the combustion reaction kinetics of AP are significantly promoted.

## Data Availability

Data will be made available on request.

## Supplementary Materials

The following supporting information can be downloaded at https://www.mdpi.com/article/10.3390/molecules31162767/s1, Section S1. Instruments; Section S2. Activation Energy Calculation; Section S3. Supplementary Figures; Figure S1. XPS test results of ZnCo-ZIF-67/CF: (a) Full spectrum; (b) C 1*s*; (c) N 1*s*; (d) O 1*s*; Figure S2. SEM image of ZnCo-ZIF-67/CF; Figure S3 EDS test results of ZnCo-ZIF-67/CF; Figure S4. TEM image of ZnCo-ZIF-67 on carbon fiber; Figure S5. TG test results of different samples in air; Figure S6. TG-DSC test results of CF-COOH + AP: (a) TG (number represents residual weight); (b) DSC; Figure S7. XRD test results of ZnCo-ZIF-67/CF-C; Figure S8. FT-IR test results of ZnCo-ZIF-67/CF-C; Figure S9. SEM/EDS test results of ZnCo-ZIF-67/CF-C; Figure S10. TG-DSC test results of ZnCO-ZIF-67/CF-C +AP: (a) TG (number represents residual weight); (b) DSC; Figure S11. Gas composition analysis of AP decomposition: (a) TG-IR diagram; (b) TG-IR mapping diagram; (c) gas composition variation over time; (d) nitrogen containing gas ratio diagram without N_2_; Figure S12. Dynamics analysis of AP: (a) TG of AP at different heating rates (numbers represent residual weight); (b) variation of activation energy of AP with reaction degree; Table S1. ICP test results of ZnCo-ZIF-67/CF; Table S2. Comparison of catalytic effects of different catalysts. References [35,36,37,38,39] are cited in the Supplementary Materials.
