# Supplementary material for: In-Situ Growth of Bimetallic ZnCo-ZIF-67 on Carbon Fibers as High-Efficiency Catalyst for Enhancing Thermal Decomposition of Ammonium Perchlorate
Source: Molecules. 2026 Aug 9;31(16):2767. doi: 10.3390/molecules31162767 (PMC13515076; doi:10.3390/molecules31162767)
Supplement: Supplementary file 1 [file molecules-31-02767-s001.zip › molecules-4427163-supplementary.pdf]

Supporting Information

Section S1. Instruments.

Fourier transform infrared spectroscopy (FT-IR)

FT-IR was recorded on a Thermo Nicolet IS50 with a diamond ATR attachments from Thermo Fisher Scientific(Waltham, Massachusetts, America).

Raman reflectance spectroscopy (Raman)

The Raman test was conducted using the RENISHAW inVia™ InSpect confocal Raman microscope from Renishaw plc (UK).

Powder X-ray diffraction (PXRD)

PXRD measurement was recorded on a Shimadzu XRD-6100 diffractometer from Japan. The diffractometer was tested under ambient temperature and pressure. The specific experimental conditions are as follows: the target material is a copper target, the wavelength of X-rays is 0.1542 nm, the scanning rate is $10^{o}\cdot min^{-1}$, and the scanning angle range is 10~80°.

X-ray photoelectron spectroscopy(XPS)

XPS was recorded on a Thermo Fisher Escalab250xi X-ray photoelectron spectrometer from Thermo Fisher Scientific(Waltham, Massachusetts, America). The light source used is an aluminum target with HV=1486.6 eV, and the analysis area is 400 μm. Before peak splitting, pollution carbon correction BE=284.8 eV is performed on the test results.

Scanning electron microscopy (SEM)

SEM images were taken using Zeiss Merlin Compact from Germany, and before shooting, the sample was treated with platinum plating to enhance its conductivity, with an acceleration voltage of 5kV.

Energy Dispersive Spectroscopy (EDS)

EDS images was conducted on the Zeiss Gemini SEM 500 from Germany with an acceleration voltage of 5kV.

Transmission electron microscopy(TEM)

TEM images were taken using a JEM-2100 transmission electron microscope, with an acceleration voltage of 200 kV. TEM elemental analysis mapping image captured on Talos F200X G2 transmission electron microscope.

ICP-AES

Into a polytetrafluoroethylene (PTFE) microwave digestion vessel was accurately weighed an appropriate amount of the sample. Subsequently added in predetermined volumes were concentrated nitric acid and hydrofluoric acid. Following sealing and placement in the rotor of the microwave digestion system, the vessel was subjected to digestion in accordance with a preset temperature program. Upon completion of the program and cooling to room temperature, carefully opened in a fume hood was the vessel. Transferred to a PTFE beaker, the digest solution was heated on a hotplate until near dryness. Upon cooling, dissolved in dilute nitric acid was the residue, which was then transferred to a volumetric flask, brought to volume with ultrapure water, and thoroughly homogenized. Prepared concurrently using the identical procedure was a sample blank.Then testing it using Agilent 5800, an inductively coupled plasma spectrometer from Agilent Technologies in the United States.

Thermogravimetric analysis

TG-DSC is used to analyze the quality changes and heat absorption and release of samples under a programmed heating system. In this article，TG-DSC analysis from 30-500 ℃ were tested on a German NETZSCH STA-2500 synchronous thermal

analyzer using a 10 °C/min^{-1} heating rate. For AP or AP/catalyst mixed samples, the testing atmosphere is nitrogen. (except for special markings).

TG-IR analysis

TG-IR testing is used to analyze the decomposition products of AP or AP/catalyst samples and for relative quantitative comparison. The carrier gas flow rate decreased to 20 mL min^{-1}. The IR instruments are Thermo Fisher and IS-10 Fourier transform infrared spectrometers.

Section S2. Activation Energy Calculation

This paper calculates the kinetics based on TG data, assuming that the total mass lost by the sample during the TG process is α=1, and then calculates α based on the mass loss at different temperatures to obtain the α - T curve. In order to further obtain the T value under a specific α, quadratic interpolation is used to process α-T.

$$f(x_0) = af_{l-1} + bf_l + cf_{l+1}$$

$$a = \frac{(x_0 - x_l) \times (x_0 - x_{l+1})}{(x_{l-1} - x_l) \times (x_{l-1} - x_{l+1})}$$

$$b = \frac{(x_0 - x_{l-1}) \times (x_0 - x_{l+1})}{(x_l - x_{l-1}) \times (x_l - x_{l+1})}$$

$$c = \frac{(x_0 - x_{l-1}) \times (x_0 - x_l)}{(x_{l+1} - x_{l-1}) \times (x_{l+1} - x_l)}$$

After obtaining the α-T curve, the E_a values of the reaction at different α values were obtained using the OZAWA method.

$$\log\beta = -\frac{0.4567\Delta E}{RT_m} - 2.315 + \log\frac{A\Delta E}{R} - \log G(x_m)$$

Section S3. Supplementary Figures

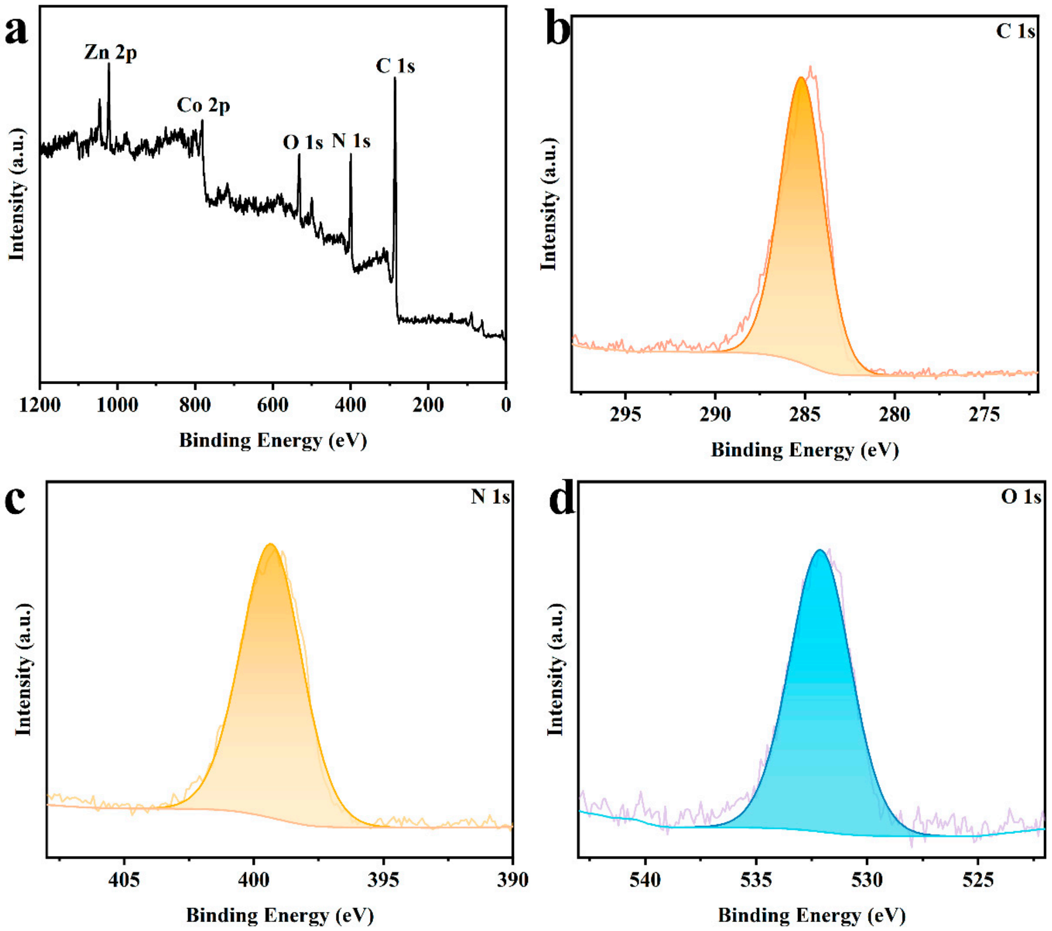

Figure S1 XPS test results of ZnCo-ZIF-67/CF:

(a) Full spectrum; (b)C 1s; (c)N 1s; (d)O 1s

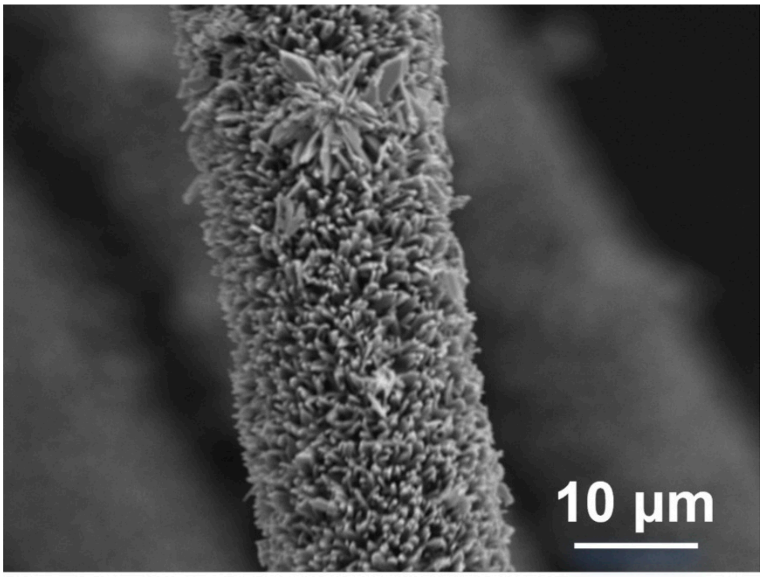

Figure S2 SEM image of ZnCo-ZIF-67/CF

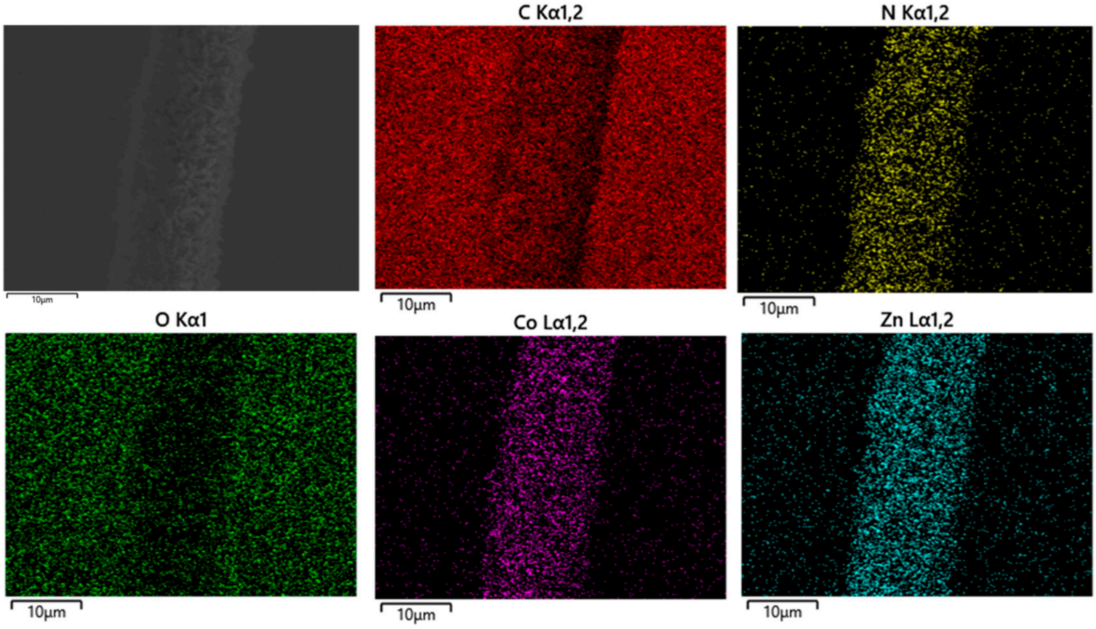

Figure S3 EDS test results of ZnCo-ZIF-67/CF

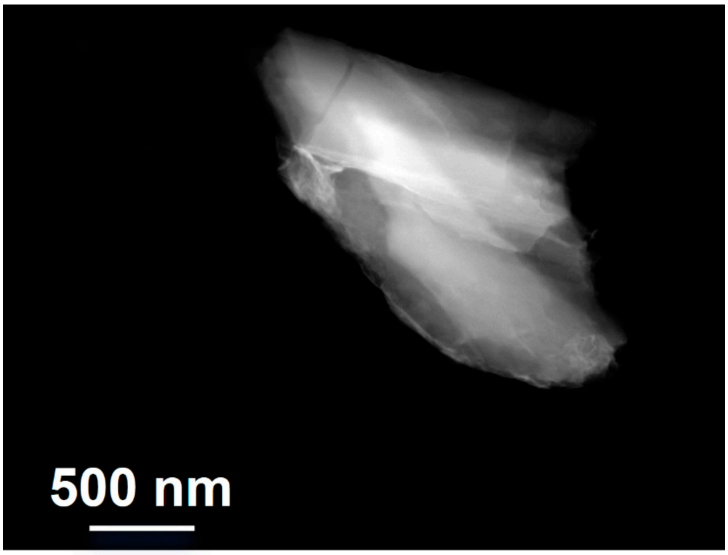

Figure S4 TEM image of ZnCo-ZIF-67 on carbon fiber

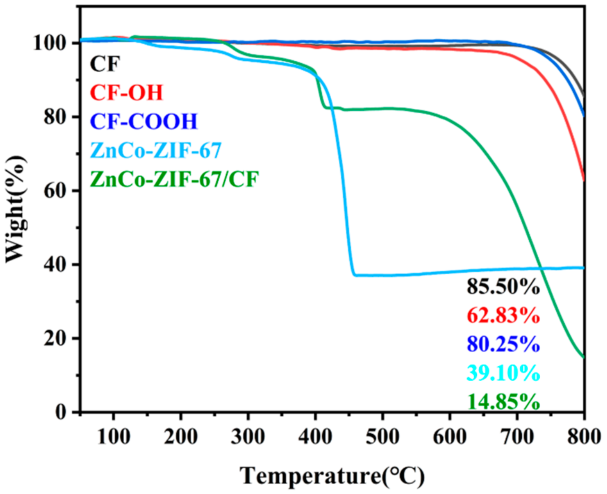

Figure S5 TG test results of different samples in air

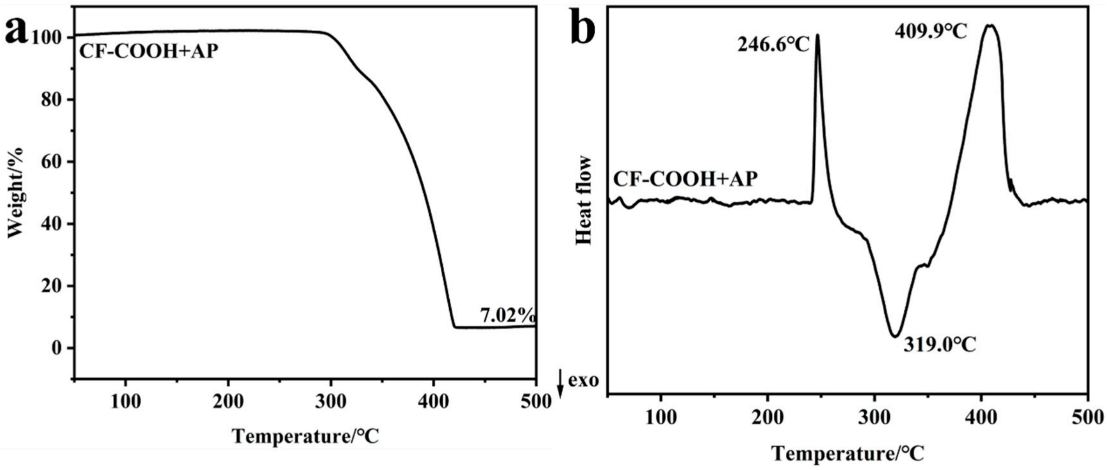

Figure S6 TG-DSC test results of CF-COOH+AP: (a) TG (number represents residual weight); (b) DSC

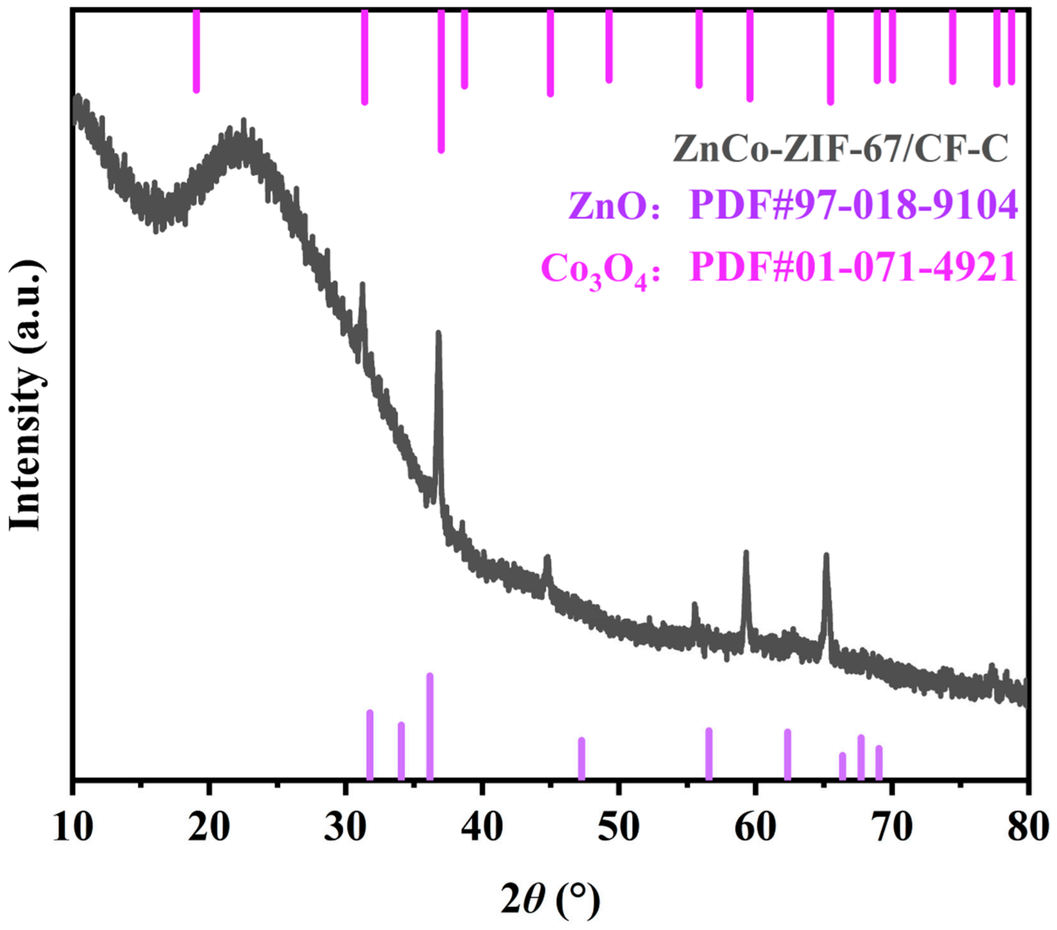

Figure S7 XRD test results of ZnCo-ZIF-67/CF-C

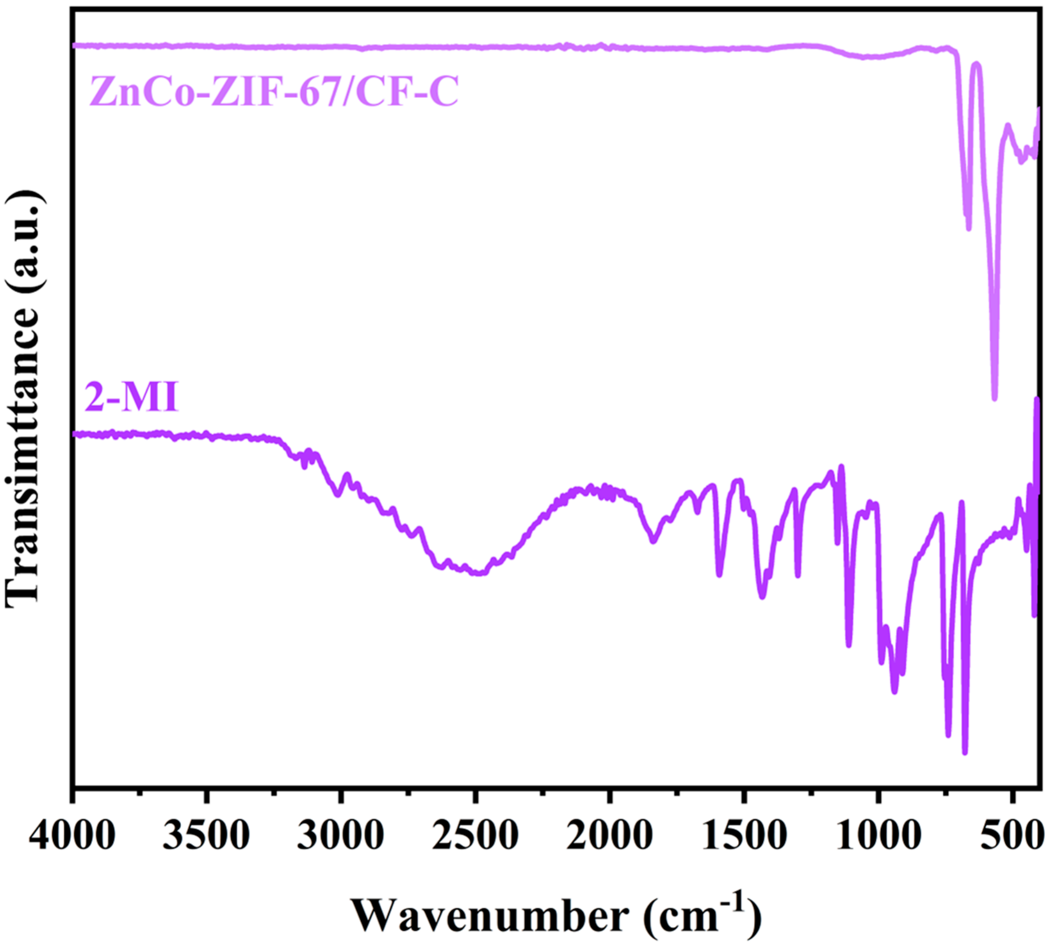

Figure S8 FT-IR test results of ZnCo-ZIF-67/CF-C

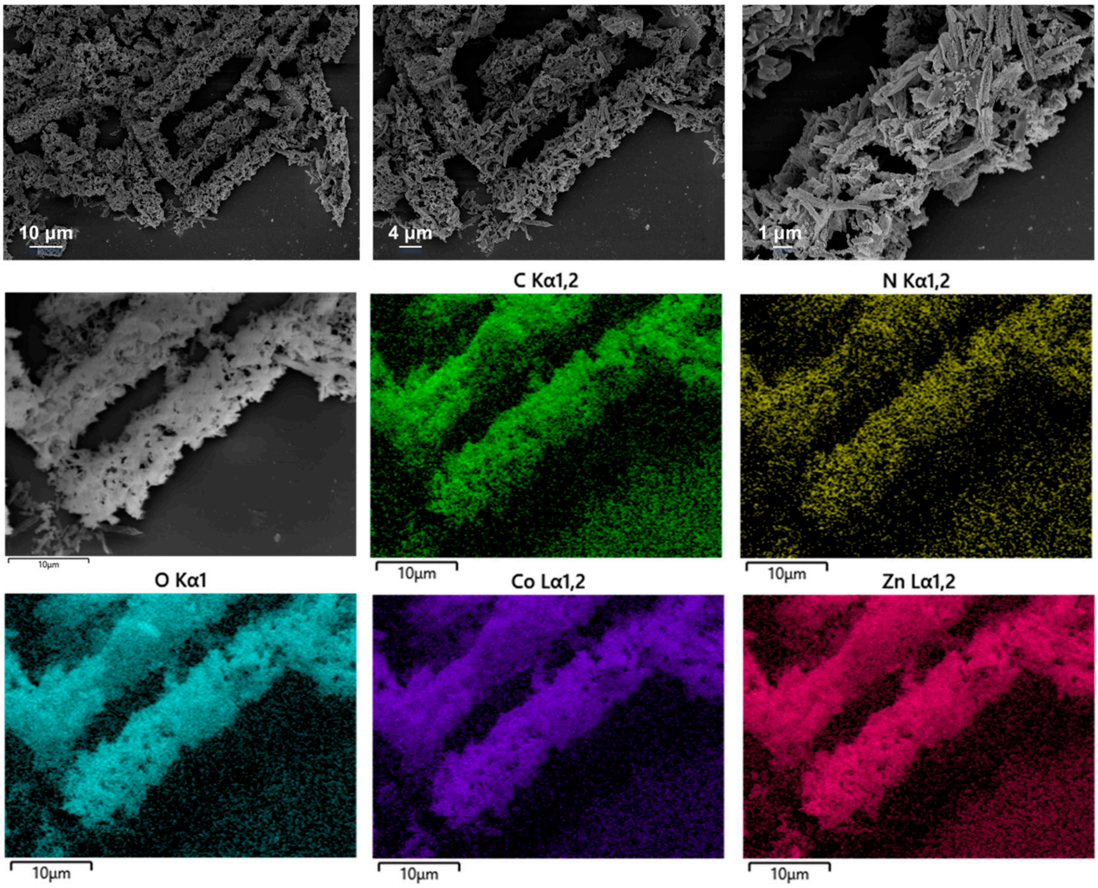

Figure S9 SEM/EDS test results of ZnCo-ZIF-67/CF-C

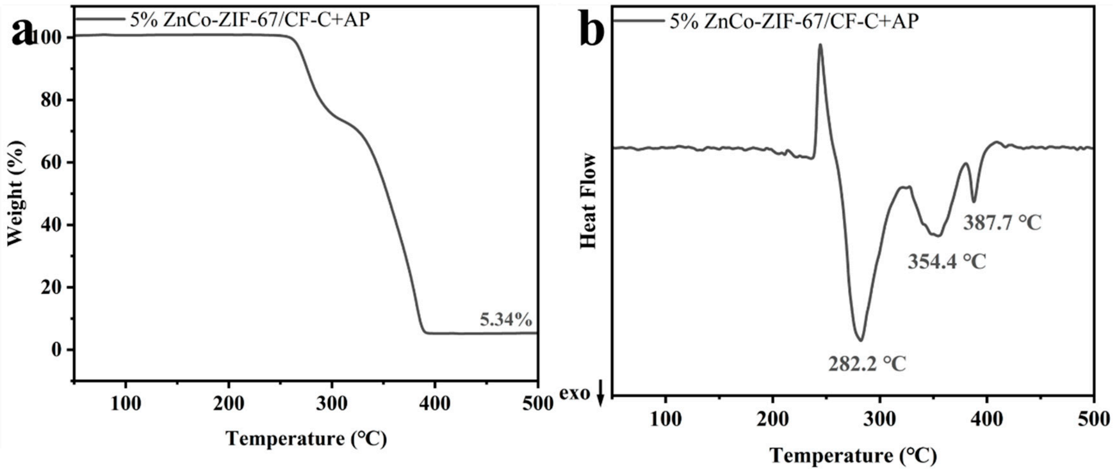

Figure S10 TG-DSC test results of ZnCO-ZIF-67/CF-C +AP: (a) TG (number represents residual weight); (b) DSC

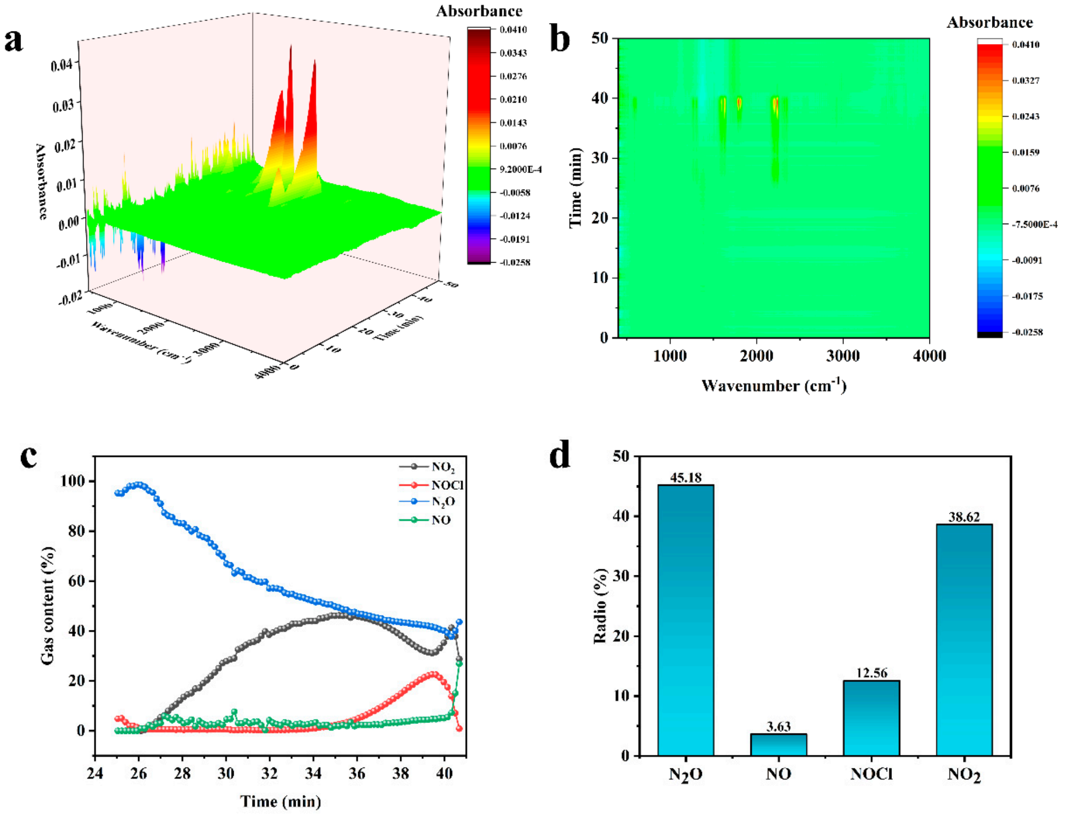

Figure S11 Gas composition analysis of AP decomposition: (a) TG-IR diagram; (b) TG-IR mapping diagram; (c) gas composition variation over time; (d) nitrogen containing gas ratio diagram without N_2

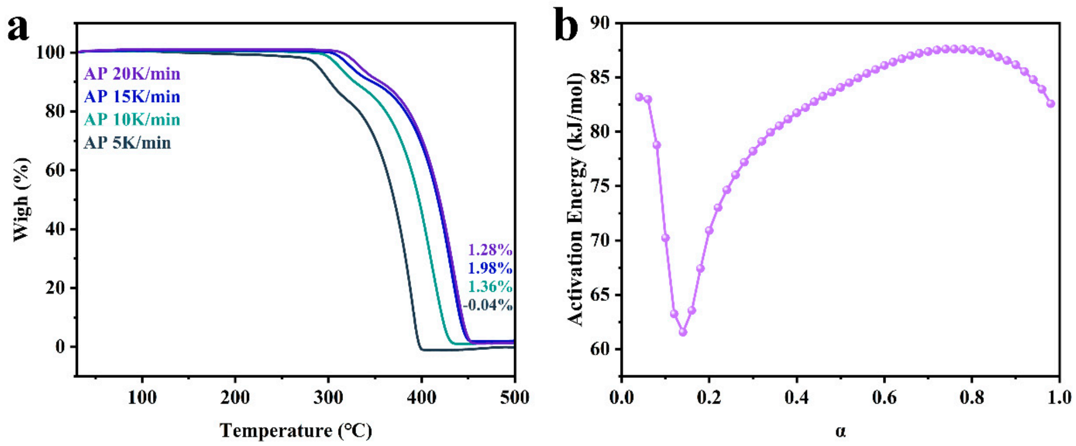

Figure S12 Dynamics analysis of AP: (a) TG of AP at different heating rates (numbers represent residual weight) (b) Variation of activation energy of AP with reaction degree

Table S1 ICP test results of ZnCo-ZIF-67/CF

Sample	Co/%	Zn/%
ZnCo-ZIF-67/CF	3.463 ± 0.007	1.293 ± 0.004

Table S2 Comparison of catalytic effects of different catalysts

Catalytic component	Additive amount/%	LTD/℃	HTD/℃	Ref
Zn-BTC	10		372.1	[S1]
Co-BTC	10		343.3	[S1]
ZnCo-BTC	10		331.8	[S1]
$[Cu(vimi)_4]DCA_2$	3		325	[S2]
$[Cu(vimi)_4]CBH_2$	3		360	[S2]
Cu-FCDT	5		346.9	[S3]
Ni-FCDT	5		374.1	[S3]
NiO-NR	4		348	[S4]
CuO-HT	3		322	[S5]
CuO-C	3		335	[S5]

In fact, the catalyst material synthesized in this paper has a relatively low effective metal content due to the presence of the carrier, and can still exhibit catalytic effects similar to those reported in previous studies, which to some extent demonstrates the excellent performance of the catalyst in this paper. In addition, the catalyst in this article plays an unparalleled role in improving the thermal conductivity of propellants compared to other catalysts.

References:

S1. Zhang, J.; Zhao, J.; Jin, B.; Peng, R. Gas−solid two-phase flow method for preparing trimesic acid series MOFs for catalytic thermal decomposition of ammonium perchlorate. *Dalton T* **2022**, 51, 17620-17628, doi:10.1039/d2dt02303b.

S2. Lei, G.; Zhong, Y.; Xu, Y.; Yang, F.; Bai, J.; Li, Z.; Zhang, J.; Zhang, T. New Energetic Complexes as Catalysts for Ammonium Perchlorate Thermal Decomposition. *Chinese J Chem* **2021**, 39, 1193-1198, doi:10.1002/cjoc.202000672.

S3. Zhao, H.; Chen, M.; Zhu, X.; Chen, S.; Bian, Z. Cu(II) and Ni(II) complexes of ferrocene-containing unsaturated β-diketones: electrochemical and burning-rate catalytic properties. *Res Chem Intermediat* **2013**, 41, 3971-3980, doi:10.1007/s11164-013-1503-7.

S4. Zhao, Y.; Zhang, X.; Xu, X.; Zhao, Y.; Zhou, H.; Li, J.; Jin, H. Synthesis of NiO nanostructures and their catalytic activity in the thermal decomposition of ammonium perchlorate. *Crystengcomm*

2016, 18, 4836-4843, doi:10.1039/c6ce00627b.

S5. Sivadas, D.L.; Thomas, D.; Haseena, M.S.; Jayalatha, T.; Krishnan, G.R.; Jacob, S.; Rajeev, R. Insight into the catalytic thermal decomposition mechanism of ammonium perchlorate. *J Therm Anal Calorim* **2019**, 138, 1-10, doi:10.1007/s10973-019-08209-5.
